# Oral Administration of Platinum Nanoparticles with SOD/CAT Cascade Catalytic Activity to Alleviate Ulcerative Colitis

**DOI:** 10.3390/jfb14110548

**Published:** 2023-11-15

**Authors:** Hao Liu, Yujie Zhang, Mingzhen Zhang, Zhaoxiang Yu, Mingxin Zhang

**Affiliations:** 1 Second Clinical Medical College, Shaanxi University of Chinese Medicine, Xianyang 712046, China; 221030111836@sntcm.edu.cn; 2 School of Basic Medical Sciences, Xi’an Jiaotong University Health Science Center, Xi’an 710061, China; zhangyujie@xjtu.edu.cn (Y.Z.); mzhang21@xjtu.edu.cn (M.Z.); 3 Department of General Surgery, The First Affiliated Hospital of Xi’an Medical University, Xi’an 710077, China; 4 Department of Gastroenterology, The First Affiliated Hospital of Xi’an Medical University, Xi’an 710077, China

**Keywords:** ulcerative colitis, platinum (Pt NPs), nanozymes, hydrogels, ROS scavenging

## Abstract

Ulcerative colitis (UC) is a refractory chronic inflammatory disease involving the colon and rectum, falling under the category of inflammatory bowel disease (IBD). The accumulation of reactive oxygen species (ROS) in local tissues has been identified as a crucial contributor to the escalation of inflammatory responses. Therefore, eliminating ROS in the inflamed colon is a promising approach to treating UC. Nanomaterials with intrinsic enzyme-like activities (nanozymes) have shown significant therapeutic potential in UC. In this study, we found that platinum nanoparticles (Pt NPs) exhibited remarkable superoxide dismutase (SOD) and catalase (CAT) cascade catalytic activities, as well as effective hydroxyl radical (•OH) scavenging ability. The in vitro experiments showed that Pt NPs could eliminate excessive ROS to protect cells against oxidative stress. In the colitis model, oral administration of Pt NPs (loaded in chitosan/alginate hydrogel) could significantly alleviate UC, including reducing the colon length, the damaged epithelium, and the infiltration of inflammatory cells. Without appreciable systemic toxicity, Pt NPs represent a novel therapeutic approach to UC and are expected to achieve long-term inflammatory remission.

## 1. Introduction

Ulcerative colitis (UC), a chronic idiopathic inflammatory bowel disease (IBD), frequently results in sustained mucosal damage spanning from the rectum to the proximal colon [1,2]. Prolonged diarrhea, abdominal pain, recurrent bloody stool, and other symptoms significantly impact the daily life of individuals with UC. In recent decades, the prevalence of UC has surged in newly industrialized nations, particularly in East Asia [3,4,5,6]. While genetic and environmental factors are commonly implicated in the etiology of UC, the precise mechanisms underlying its pathogenesis remain elusive [7,8]. Combined treatment with chemical and biological agents such as 5-aminosalicylic acid (5-ASA), cortisol, and calcitonin inhibitors is the predominant approach to managing UC [9,10]. However, long-term, high-dose chemotherapy drugs frequently result in severe complications, including infection or thrombosis, necessitating surgical intervention for some UC patients due to complications such as intestinal stenosis or fistula [11,12,13]. Consequently, developing an innovative treatment for IBD is significant in addressing clinical challenges.

Prior research has demonstrated that a considerable quantity of inflammatory cells aggregate within the colon of individuals with inflammatory bowel disease (IBD), leading to the generation of reactive oxygen species (ROS), including hydrogen peroxide (H_2_O_2_), hydroxyl radical (•OH), and superoxide anion (O_2_•−) [14,15]. The resultant oxidative stress caused by ROS instigates harm to the intestinal mucosa, thereby compromising cellular structures such as the intestinal cell membrane, protein, and DNA, consequently exacerbating the advancement of IBD [16]. Therefore, using antioxidant enzymes to eliminate surplus ROS within the inflammatory microenvironment proves highly advantageous in upholding intestinal redox equilibrium and effectively managing UC [17]. Natural enzymes have high substrate specificity and catalytic activity. Still, their high cost and low stability have become the primary intrinsic defects that limit their wide application in the medical field [18,19].

In recent times, considerable scholarly focus has been directed toward artificial enzymes, which can imitate the functionality and specificity exhibited by natural enzymes. These artificial nanozymes offer a cost-effective alternative and address the inherent instability associated with natural enzymes. Consequently, they hold significant promise for application in disease diagnosis and treatment [20,21].

Platinum nanoparticles (Pt NPs) are emerging as a new class of nanozymes with distinct side effects and unique electronic structures, and these properties endow Pt NPs with potent superoxide dismutase (SOD) and catalase (CAT) activities, which can cascade catalyze O_2_•− decomposition [22]. Previous studies have demonstrated the successful synthesis of Pt NPs (30 nm) as antioxidant agents for preventing and treating hepatic ischemia-reperfusion injury [23]. Moreover, Pt NPs have shown potential as antioxidant drugs for atherosclerosis by effectively eliminating the ROS produced by vascular endothelial cells under high glucose and lipid level conditions [24]. Therefore, the Pt-NP-based treatment of UC is a promising strategy for disease management.

Polyvinylpyrrolidone (PVP) is a kind of non-toxic, non-ionic polymer, and its unique chemical structure allows it to be used as a stabilizer to prevent the aggregation of nanoparticles in water or other organic solvents during the preparation of nanomaterials [25]. We considered the synthesis of Pt NPs using PVP. Chitosan/sodium alginate hydrogels, known for their excellent biocompatibility and biodegradability, are commonly used as carriers for nanomaterials [26,27]. Hydrogels enable drug delivery and sustained release within the gastrointestinal tract by loading anti-inflammatory or immunomodulatory drugs, enhancing therapeutic efficacy at inflammatory sites. It is worth mentioning that chitosan exhibits adhesive properties in the intestinal mucosa, further enhancing the drug concentration at sites of inflammation [28]. Therefore, selecting hydrogels for encapsulating Pt NPs holds promise for treating UC.

**Figure 1:**
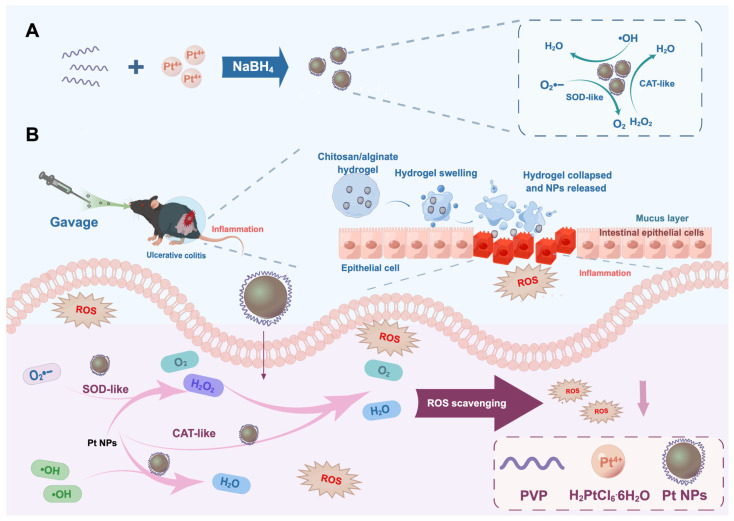
Schematic of the synthesis of Pt NP nanozymes and oral administration of Pt NPs encapsulated in chitosan/sodium alginate hydrogel to alleviate ulcerative colitis. (**A**) Synthesis of Pt NPs with enhanced SOD and CAT activities and •OH scavenging capacity via reduction method. (**B**) Oral administration of Pt NPs encapsulated in chitosan/sodium alginate hydrogel to alleviate ulcerative colitis.

In this study, we synthesized Pt NPs with a reduced particle size to evaluate their therapeutic potential for UC. We mixed chloroplatinic acid hexahydrate (H_2_PtCl_6_·6H_2_O) with PVP to prepare small-particle-size Pt NPs via reduction (Figure 1). To achieve targeted therapy and complete the construction of the nano-delivery system, we further loaded Pt NPs into a PH-responsive chitosan/alginate hydrogel. Upon oral administration, the hydrogel swells and ruptures in the alkaline environment of the colon, facilitating the release of Pt NPs. The internalized Pt NPs effectively eliminate ROS at the inflamed sites within colonic epithelial cells, thereby preventing oxidative damage to the intestinal epithelium. The oral administration of Pt NPs loaded within the chitosan/alginate hydrogel offers a novel strategy for alleviating UC inflammation.

## 2. Materials and Methods

### 2.1. Materials and Instruments

The chloroplatinic acid hexahydrate (H_2_PtCl_6_·6H_2_O), terephthalic acid (TA), and polyvinylpyrrolidone (PVP) were purchased from Aladdin Technology Co., Ltd. (Shanghai, China). The sodium borohydride (NaBH_4_) was obtained from Sinopharm Group Chemical Reagent Co., Ltd. (Shanghai, China). The nitroblue tetrazolium chloride (NBT), L-methionine (L-met), and riboflavin were all sourced from Sigma-Aldrich (Shanghai, China). The hydrogen peroxide (H_2_O_2_, 30%) was obtained from Beijing Chemical Reagents Company (Tianjin, China). The methylene blue (MB) was purchased from Chemical Reagent Co., Ltd. (Tianjin, China). The ferrous chloride dihydrate (FeCl_2_·4H_2_O) was acquired from Bioren Biological Technology Co., Ltd. (Tianjin, China). The 2,7-dichlorofluorescein diacetate (DCFH-DA) was sourced from Beyotime Biotechnology (Shanghai, China). The chitosan was acquired from Tokyo Chemical Industry Co, Ltd. (Tokyo, Japan), the sodium alginate from Aladdin Co, Ltd. (Shanghai, China), and the 5-aminosalicylic acid (5-ASA) from Sangon Biotech Co., Ltd. (Shanghai, China).

The primary experimental equipment utilized in the study included a transmission electron microscope (JEOL JEM-2100Plus, Tokyo, Japan), X-ray diffractometer (Bruker D8 ADVANCE, Bruker, Billerica, MA, USA), ultraviolet absorption spectrophotometer (UV-2700, Shimadzu Corporation, Kyoto, Japan), Malvern particle size analyzer (Nano ZSE, Malvern, Shanghai, China), dissolved oxygen meter (JPSJ-605F, Leybold, Shanghai, China), and fluorescence spectrophotometer (Hitachi F-4700, Hitachi, Tokyo, Japan).

### 2.2. Preparation and Characterization of Pt NPs

The Pt nanoparticles were synthesized via chemical reduction. Respectively, 86 mg of PVP and 27.13 mg of H_2_PtCl_6_·6H_2_O were dissolved in 51.4 mL and 8.6 mL of water. The two solutions were mixed. Subsequently, 1 mL of NaBH_4_ solution (10 mg/mL) was added dropwise under continuous stirring. The solution exhibited a color change from yellow to dark brown and generated many bubbles. After completion of stirring, the solution was allowed to stand undisturbed for 24 h, followed by rotary evaporation for 15 min to reduce the solution volume. Water dialysis was conducted for 24 h to remove the NaBH_4_ residues, followed by subsequent rotary evaporation to obtain a precipitate of the Pt colloid. The deposit was then treated with 3 mL of acetone and subjected to sonication, after which rotary evaporation was applied to remove the residual acetone. A volume of 2 mL of ethanol was introduced into the sediment and subjected to sonication for a duration (15 min). The resulting mixture was blended and subsequently subjected to centrifugation (3000 rpm/10 min). The washing process was repeated three times using ethanol and hexane to ensure the removal of excess PVP. Finally, the Pt NPs synthesis was concluded by resuspending the residue in 2 mL of water.

The total Pt content in the aqueous Pt NPs was determined using inductively coupled plasma atomic emission spectrometry (ICP-AES). The morphology and size of the Pt NPs were characterized using transmission electron microscopy (TEM). X-ray diffraction (XRD) analysis was utilized to ascertain the crystal structure of the Pt nanoparticles. The optical properties of Pt NPs were evaluated by measuring the ultraviolet-visible (UV-Vis) spectra using an ultraviolet absorption spectrophotometer. The stability of the Pt NPs was assessed by measuring their zeta potential using a Malvern ZS90 particle size analyzer.

### 2.3. SOD-like Activity of Pt NPs

The SOD-like activity of the Pt NPs was tested using the NBT reduction method. Riboflavin undergoes reduction when exposed to light, followed by subsequent oxidation in the presence of oxygen to generate O_2_•−. This reactive oxygen species then reacts with NBT to form a blue methyl hydrazone compound, which exhibits its maximum absorption at 560 nm. The nanoparticles (NPs) possessing superoxide dismutase (SOD) activity effectively scavenge O_2_•−, thereby inhibiting the formation of methyl hydrazone. The experimental setup constructed a reaction system by mixing L-MET (13 mM), riboflavin (20 μM), NBT, and PBS buffer. Subsequently, Pt NPs with varying concentrations (0–100 μg/mL) were added. Following continuous illumination for 2 min, the absorption at 560 nm was quantified using a microplate reader. 

### 2.4. CAT-like Activity of Pt NPs

The CAT activity of the Pt NPs was measured using the dissolved oxygen method. CAT enzyme catalyzes the decomposition of H_2_O_2_ to produce oxygen. Different concentrations (0.1, 0.2, 0.4, 0.8, 1, 1.5 μg/mL) of Pt NPs were added to a 10 mL ultrapure water system containing the same concentration of H_2_O_2_. The dissolved oxygen meter was adjusted to the kinetic mode to monitor the changes in oxygen content (mg/L) in the reaction solution. Using the same method, equal amounts of Pt NPs were introduced into a 10 mL ultrapure water system containing different concentrations of H_2_O_2_ (200 μM, 1 mM, and 2 mM) to study the substrate concentration dependence of the CAT enzyme activity of the Pt NPs.

Michaelis–Menten constant determination was performed by setting up different concentrations of H_2_O_2_ (0.5, 1, 2, 4, 8, 16, 32, 64, 96, 128, and 144 mM) in a 10 mL ultrapure water reaction system. An equal amount of Pt NPs with a concentration of 0.175 μg/mL was added to the system. The oxygen concentration (mg/L) was subsequently measured using a dissolved oxygen meter, and the measurements were concluded once the oxygen concentration reached equilibrium.

### 2.5. •OH Scavenging Capacity Test

We validated the •OH scavenging ability of aqueous solutions of Pt NPs using two methods.

Method One: Methylene blue (MB) fading strategy. The absorption peak of MB in the visible range is at 664 nm. The •OH generated by Fenton’s reagent can cause the fading of MB, leading to a reduction in its absorption at 664 nm. Pt NPs at a concentration of 25 μg/mL were mixed with 1 mM Fe^2+^ and 5 mM H_2_O_2_ in PBS buffer (25 mM, pH 7.4), and the mixture was incubated at 37 °C for 15 min. Subsequently, 0.25 mM MB was added, and the absorbance of MB at 664 nm was measured using a UV spectrophotometer.

Method Two: Terephthalic acid (TA) method. Ultraviolet (UV) radiation can induce the production of a large amount of •OH from H_2_O_2_, and •OH readily reacts with TA to form 2-hydroxyterphenylacid, which exhibits fluorescence properties (excitation wavelength: 320 nm, emission wavelength: 425 nm). After irradiation of the PBS buffer containing H_2_O_2_ (5 mM) and TA (0.5 mM) under UV light for 15 min, different concentrations of Pt NPs (12.5, 25, 50, 100 μg/mL) were added, and the fluorescence intensity of the mixture was measured using a fluorescence spectrophotometer.

### 2.6. Anti-Oxidative Effect of Pt NPs in Living Cells

The RAW 264.7 cells were purchased from Procell Co., Ltd (Wuhan, China). The RAW 264.7 cells were evenly distributed in 6-well plates at 5 × 10^3^ cells/well density and incubated overnight at 37 °C. The Pt NP solution (50 μg/mL) was prepared using Dulbecco’s Modified Eagle’s Medium (DMEM) and incubated for an additional 8 h. The medium was removed, and the cells were treated with a solution of a ROS-positive control, named Rosup (0.5 mg/mL), for 30 min to induce the production of ROS. The cells were stained using 10 μM of 2, 7-dichlorofluorescein diacetate (DCFH-DA) and examined using a fluorescence microscope.

### 2.7. Preparation of Chitosan/Alginate Hydrogel

The chitosan/alginate saline gel was prepared according to the relevant literature [29,30,31]. Chitosan powder was dissolved in acetic acid, and the pH was adjusted to approximately 7.0 using a NaOH solution (0.15 mol·L^−1^). The resulting solution was then scaled to a final concentration of 0.6% (wt./vol) and stirred overnight at room temperature. Sodium alginate was dissolved in a 0.15 M NaCl solution, and the final concentration was adjusted to 1.4% (wt./vol). The chitosan and sodium alginate solutions were mixed in a 1:1 ratio, and the nanoparticles were added. The chelating solution, containing 30 mM Na_2_SO_4_ and 140 mM CaCl_2_, was prepared at a 1:1 volume ratio. Before utilization, the polysaccharide solution and the chelating solution containing the nanoparticles underwent preheating at 37 °C for 30 min.

### 2.8. Animals

The present study was approved by the Animal Ethics Committee of Xi’an Jiaotong University (ethical protocol approval n. XJTUAE2023-1564). Female C57/BL6 mice (8 weeks old) were obtained from the Animal Center of Xi’an Jiaotong University Health Science Center, Shaanxi province, China. The mice were housed under standard laboratory conditions, following the animal care and use guidelines.

### 2.9. Colitis Model of Mice

Eight-week-old female C57 mice were randomly assigned to one of four groups: a healthy group, a DSS group, a DSS + 5-ASA group, and a DSS + Pt NPs group. Except for the healthy group, all mice received ad libitum administration of 2.5% *w*/*v* dextran sulfate sodium (DSS; 36–50 Kda) solution for seven days to induce acute UC. Throughout the modeling period, the mice in the treatment groups received gavage treatment on the 2nd, 4th, and 6th day. Daily weight changes, hematochezia, and disease activity index records were kept. On the 8th day, all mice were euthanized, and their distal colon tissues were collected for subsequent analysis.

### 2.10. Statistical Analysis

The results were analyzed using a one-way ANOVA test and Student’s *t*-test. Statistical significance: * *p* < 0.05, ** *p* < 0.01, *** *p* < 0.001.

## 3. Results

### 3.1. Synthesis and Characterization of Pt NPs

**Figure 2:**
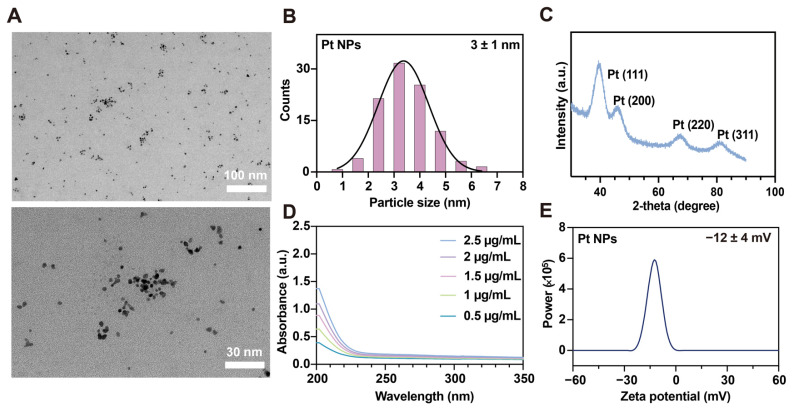
Characterization studies of Pt NPs. (**A**) TEM images of Pt NPs. Scale bar: 100 nm; 30 nm. (**B**) Particle size distribution of Pt NPs. N = 126. The solid black line represents the fitted distribution function: Y = 32.32 × exp(−0.5 × ((X − 3.369)/0.9812)^2^). (**C**) X-ray powder diffraction pattern of Pt NPs. (**D**) UV-vis absorption spectra of Pt NPs with different concentrations. (**E**) Zeta potential of Pt NPs.

Firstly, the Pt NPs were synthesized using a reduction method [32]. The morphology of the prepared Pt NPs was observed using TEM, revealing a uniform and stable distribution in the aqueous solution (Figure 2A) [33]. The size distribution histogram of the Pt NPs, mounted using the Sturges method, revealed an average particle diameter size of 3 ± 1 nm (Figure 2B) [34]. The XRD technique was employed to determine the crystal structure of Pt NPs. The results illustrated the distinct diffraction peaks observed at 2θ values of 39.9°, 46.1°, 68.1°, and 81.5°, which corresponded to the 111, 200, 220, and 311 planes of the Pt crystal morphology, respectively (Figure 2C). All these results provided evidence for the successful synthesis of the Pt NPs.

The presence of an absorption peak in the H_2_PtCl_6_ solution is attributed to the PtCl_6_^2−^ ions. In specific sizes and shapes, such as nanoscale surface coatings, platinum nanoparticles exhibit a visible absorption peak within the UV spectrum due to the surface plasmon resonance effect. However, conventional platinum nanoparticle colloidal solutions usually lack absorption peaks in the UV spectrum range. This study’s UV-visible spectroscopic analysis of Pt NPs with varying concentrations confirmed the absence of absorption peaks within the UV spectrum range, consistent with previous research findings (Figure 2D) [35,36,37]. Colitis frequently results in mucosal layer impairment, causing an accumulation of positively charged molecules in the affected epithelial region. Consequently, negatively charged nanomaterials have the potential to explicitly target and treat inflamed epithelia [38]. The zeta potential technique serves as an indirect means of assessing the surface charge of nanoparticles. The stability of Pt NPs was evaluated by measuring the zeta potential [39]. The results reveal that the zeta potential of Pt NPs in an aqueous solution was −12 ± 4 mV (Figure 2E). The characteristic negative charge promisingly facilitated the rapid targeting of Pt NPs to sites of inflammation, thereby enhancing the therapeutic effectiveness.

### 3.2. ROS Scavenging Ability of the Pt NPs

**Figure 3:**
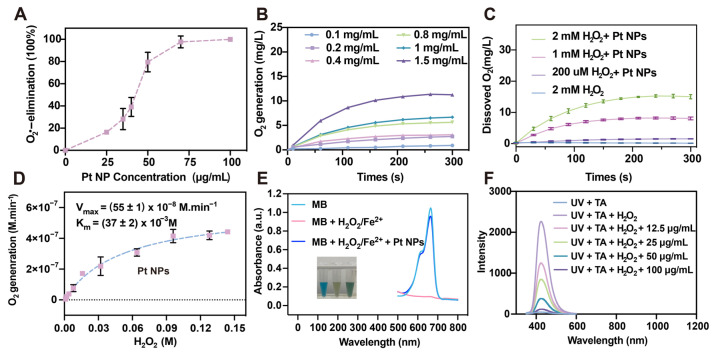
ROS scavenging capacity of the Pt NPs. (**A**) Percentage of O_2_^•−^ elimination by Pt NPs in various concentrations via the NBT method. (**B**) Dissolved oxygen production from H_2_O_2_ under different Pt NP concentrations. (**C**) CAT-like enzyme activity of Pt NPs (1.75 μg/mL) under varied H_2_O_2_ concentrations. (**D**) The Michaelis–Menten kinetics assay for the CAT-like enzyme activity of the Pt NPs (with 0.175 μg/mL Pt NPs and 0–0.144 M H_2_O_2_). (**E**) MB method determined the •OH scavenging ability of the Pt NPs (20 μg/mL). Inset: Images of different reaction systems. (**F**) TA method determined the •OH scavenging ability of the Pt NPs.

The antioxidant capabilities of nanomaterials are crucial for an anti-inflammatory therapeutic role. Hence, the antioxidant activity of the Pt NPs was further evaluated. The NBT method was utilized to examine the SOD-like activity of the Pt NPs. The results revealed that the SOD activity of the Pt NPs increased with rising concentrations, and the Pt NPs eliminated O_2_•− at a concentration of 100 μg/mL (Figure 3A). The SOD enzyme facilitates the dismutation of O_2_•− to generate H_2_O_2_, which possesses oxidizing properties, and the CAT enzyme can catalyze H_2_O_2_ into non-toxic oxygen and water. The dissolved oxygen method was employed to measure the CAT-like enzyme activity of the Pt NPs. The findings indicated that Pt NPs exhibited significant efficacy in catalyzing oxygen production from H_2_O_2_. At room temperature, as the concentration of Pt NPs increased, the volume of oxygen produced also escalated (Figure 3B). Moreover, under the equivalent concentration of Pt NPs and varying concentrations of H_2_O_2_, Pt NPs exhibited substrate-concentration-dependent CAT-like enzyme activity (Figure 3C). The Michaelis–Menten kinetics were calculated, and it was determined that the *K_M_* of Pt NPs was (37 ± 2) × 10^−3^ M and the *V_max_* was (55 ± 1) × 10^−8^ M·min^−1^, which suggested that the Pt NPs demonstrated a good affinity for H_2_O_2_ (Figure 3D). •OH is another highly reactive oxidative ROS, and excessive •OH in human cells often inflicts severe oxidative damage on the DNA structure [40]. To assess the efficacy of Pt NPs in eliminating •OH, MB was employed as an indicator to detect •OH. The methylene blue (MB) solution is blue, with an absorption peak at 664 nm in the visible range. The •OH generated by Fenton’s reagent causes the bleaching of the MB solution, resulting in a decrease in its absorption peak at 664 nm. The results showed that a single MB solution exhibited a distinct absorption peak at 664 nm, and the solution appeared blue. Introducing Fenton’s reagent lightened the MB solution color and nearly eradicated the absorption peak at 660 nm. However, when Pt NPs were added to the mixture, the absorption peak at 660 nm was restored, indicating that Pt NPs can effectively eliminate •OH (Figure 3E). Furthermore, the efficacy of the Pt NPs in eliminating •OH was indirectly assessed by employing terephthalic acid (TA). Ultraviolet (UV) radiation can induce the production of a large amount of •OH from H_2_O_2_, and •OH readily reacts with TA to form 2-hydroxyterphenylacid, which exhibits fluorescence properties (excitation wavelength: 320 nm; emission wavelength: 425 nm). The experimental findings showed that the UV-irradiated TA mixture had an absorption peak at 425 nm, while the absorption peak of the TA mixture diminished after adding Pt NPs (Figure 3F). In addition, the extent of reduction in the absorption peak is proportional to the concentration of Pt NPs (Figure 3F). The observation suggested that Pt NPs eliminated the •OH production and thus reduced the production of the fluorescent compound 2-hydroxyterephthalic acid.

### 3.3. The Intracellular ROS Scavenging Capacity of Pt NPs

**Figure 4:**
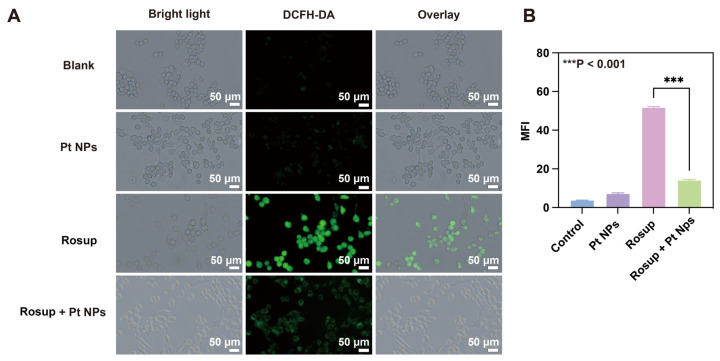
In vitro anti-oxidative of Pt NPs. (**A**) Characteristic fluorescence images of RAW 264.7 cells stained using DCFH-DA after various treatments. Scale bar: 50 μm. (**B**) Mean fluorescence intensity of RAW 264.7 cells stained using DCFH-DA. *** *p* < 0.001.

The large amount of ROS in the intestinal epithelial cells of IBD patients is closely related to the aggregation and activation of inflammatory cells (such as neutrophils) in the inflammatory site. The synergistic effect of the ROS and inflammatory cells leads to cell damage and oxidative stress, ultimately worsening the inflammatory microenvironment and exacerbating colitis symptoms [41]. Consequently, the elimination of excessive ROS within cells holds immense importance in the management of UC. The antioxidant potential of Pt NPs was examined at the cellular level. As a fluorescent probe, 2′, 7′-dichlorofluorescein diacetate (DCFH-DA) was employed to detect intracellular ROS. DCFH-DA possesses the ability to permeate the cell membrane and react with intracellular ROS, resulting in the production of 2′, 7′-dichlorofluorescein (DCF) with green fluorescence emission. By measuring the fluorescence intensity, the level of intracellular ROS could be indirectly evaluated [42]. Rosup was an exogenous inducer to elicit ROS in RAW 264.7 cells (macrophages typical in inflammatory responses), with a DCFH-DA probe for measuring the intracellular ROS levels. The results indicated that the control group and the group treated with Pt NPs exhibited negligible fluorescence in the RAW 264.7 cells, meaning that the cells had almost no ROS. The fluorescence intensity significantly increased in the cell group stimulated using Rosup, and after co-incubating with Pt NPs, a notable decrease in green fluorescence was observed, suggesting that Rosup effectively induced ROS production, and Pt NPs significantly reduced ROS. (Figure 4A). The conclusion was confirmed via statistical analysis of the fluorescence intensity (Figure 4B).

### 3.4. In Vivo Determination of the Biocompatibility of Pt NPs

**Figure 5:**
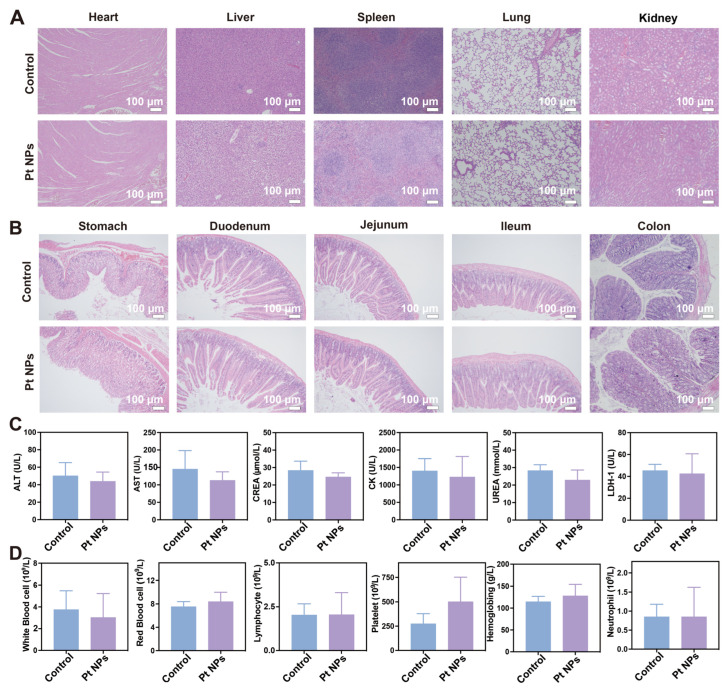
Biocompatibility evaluation of Pt NPs. (**A**) Histological assessment of major organs using HE staining to examine the systemic toxicity of Pt NPs. Scale bar: 100 μm. (**B**) HE staining evaluation of Pt NP toxicity in the digestive system. Scale bar: 100 μm. (**C**) Analysis of complete blood cell counts in mice treated using Pt NPs. (**D**) Analysis of blood biochemistry parameters in mice treated using Pt NPs.

Nanomaterials used in biomedicine come into direct contact with blood and tissues when introduced into the human body, potentially causing toxic side effects [43,44]. Consequently, an animal experiment was devised to assess the biocompatibility of Pt NPs. A cohort of healthy mice was randomly allocated into two groups, wherein the treatment group was administered Pt NPs every alternate consecutive duration of 7 days. After 14 days, the mice were euthanized, and their organs and various gastrointestinal tract segments were collected for histological examination using hematoxylin and eosin staining. Blood samples were obtained from both groups of mice for biochemistry analysis and complete blood cell count (CBC). A comparison of the H and E staining results of the vital organs between the control and treatment groups did not reveal any significant differences, indicating no observable tissue damage in either group (Figure 5A). Specifically, the liver displayed a typical arrangement of hepatocytes without any evidence of necrotic edema. The heart exhibited intact myocardial fibers and no cellular vacuolation. The histological analysis of the gastrointestinal tract tissues (including the stomach, duodenum, jejunum, ileum, and colon) obtained from mice treated using Pt NPs demonstrated comparable tissue morphology to the control group. No evidence of intestinal epithelial detachment, defects, or the infiltration of inflammatory cells was observed in either group (Figure 5B). The blood biochemical indexes of mice in both the administration group and the healthy group were assessed, encompassing alanine aminotransferase (ALT), aspartate aminotransferase (AST), creatinine (CREA), urea, creatine kinase (CK), and lactate dehydrogenase-1 (LDH-1). The findings indicated that all biochemical indexes in both groups were within the established normal range (Figure 5C).

Consequently, it could be inferred that Pt NPs did not induce significant harm to vital organs such as the liver, kidneys, and cardiac muscle in mice. CBC analysis showed no significant changes in the red blood cells, white blood cells, neutrophils, lymphocytes, platelets, and hemoglobin levels between the treatment group and the control group (Figure 5D). Consequently, based on these experimental findings, it can be inferred that orally administered chitosan/alginate-coated Pt NPs demonstrate favorable biocompatibility.

### 3.5. Protective Effect of Pt NPs against Colitis

**Figure 6:**
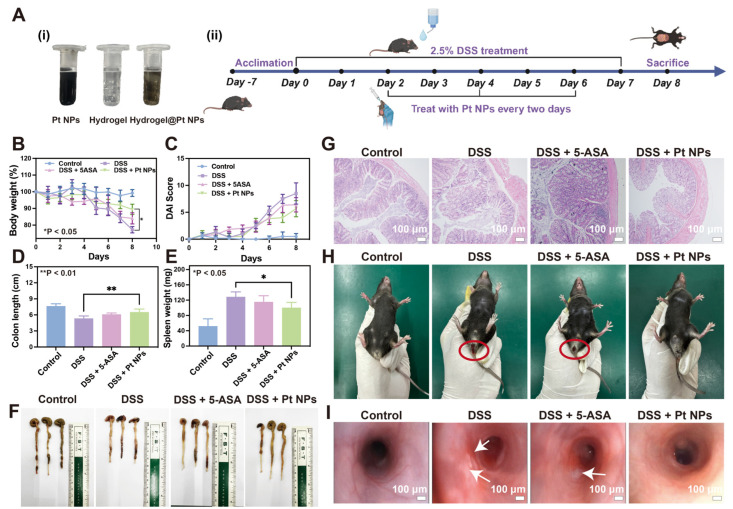
Protective effect of Pt NPs in the DSS-induced colitis model. (**A**) Diagram illustrating the application of Pt NPs in the treatment of DSS-induced colitis mice. (**B**) Weight changes in mice over 8 days. (**C**) Changes in DAI scores of mice over 8 days. (**D**) Assessment of colon length in mice subjected to different treatments. (**E**) Assessment of spleen weight in mice subjected to different treatments. (**F**) Photographs of excised colons from mice in different treatment groups showing differences in length. (**G**) HE-stained histological analysis of colonic tissues from mice subjected to different treatments on day 8. Scale bar: 100 μm. (**H**) Representative photographs of rectal bleeding in the different groups. (**I**) Endoscopic images of the colon in the different groups. Scale bar: 100 μm. * *p* < 0.05, ** *p* < 0.01.

The protective effect of Pt NPs on the colon was investigated in a UC mouse model induced by dextran sodium sulfate (DSS). Oral administration is preferred for ulcerative colitis treatment due to its simplicity, convenience, and safety [45]. The Pt NPs were encapsulated within a hydrogel composed of chitosan and sodium alginate for oral administration (Figure 6A, i). The hydrogel exhibited a tight structure in the gastric acid environment, preventing premature release of the Pt NPs. Nevertheless, upon arrival at the location of colonic inflammation, the alkaline surroundings prompted an escalation in the swelling characteristics of chitosan, resulting in the expansion of the hydrogel and subsequent liberation of the Pt NPs. The methodology accomplished targeted treatment of colonic inflammation via Pt NPs [46]. A 2.5% DSS solution was employed over 8 days to simulate acute UC in mice (Figure 6A, ii). In the treatment group, dosing was administered on days 2, 4, and 6. To evaluate the efficacy of the Pt NPs, 5-aminosalicylic acid (5-ASA) was employed as a positive control for treating UC. The body weight of the mice in different groups was monitored during the modeling and treatment period, reflecting the effects of modeling and treatment. The findings indicated that compared to the healthy group, mice subjected to DSS induction experienced weight reduction from day 5 (Figure 6B).

Meanwhile, mice treated with oral 5-ASA and Pt NPs showed a significantly milder decrease in body weight (Figure 6B). Furthermore, mice treated with Pt NPs exhibited a significantly lower disease activity index (DAI) than the DSS group (Figure 6C). As a vital immune organ, the weight change in the spleen can be used as an index to evaluate the level of inflammation [47]. The spleen of the mice was weighed after execution. The spleen weight of the mice treated with Pt NPs was significantly lower than that of the DSS group, indicating effective prevention of inflammation by Pt NPs (Figure 6D). The colon length of mice induced using DSS was significantly reduced compared with that of healthy mice, while adding Pt NPs impeded this reduction in colon length (Figure 6E,F). Subsequently, the colon tissues of the mice were stained using hematoxylin–eosin (Figure 6E,H). The results showed the integrity of the colon epithelial cells in mice induced using DSS was destroyed, and many inflammatory cells infiltrated the intestinal epithelium, which was evidence of the inflammatory response, leading to cell damage and death [48]. The colonic continuity of mice with 5-ASA and Pt NP treatment remained largely intact, had no apparent instances of rupture, and exhibited a notable reduction in the infiltration of inflammatory cells (Figure 6G). Anal bleeding is a manifestation of extensive damage to the intestinal mucosa. During treatment, blood in the stool was observed in mice, particularly those with induced UC via DSS administration. However, mice treated with oral Pt NPs exhibited a reduction in the symptoms of anal bleeding (Figure 6H). Endoscopy is the preferred method for clinically diagnosing UC, offering precise lesion localization and evaluation of disease activity.

Moreover, it plays a crucial role in guiding the modification and implementation of treatment strategies for intestinal diseases [49,50]. The endoscopic results demonstrated that Pt NP treatment significantly reduced colon mucosal congestion, edema, and ulceration in the UC mouse model (Figure 6I). These results all supported that Pt NPs effectively prevent colitis.

## 4. Discussion

UC has historically been characterized as a chronic inflammatory disorder resulting from an aberrant immune response. As the main symptoms of UC, recurrent bloody stool and diarrhea seriously affect the average life of patients [51]. Prolonged exposure to inflammatory factors can exacerbate colonic mucosal damage in certain UC patients, frequently leading to the development of inflammatory colon cancer [52,53]. In the clinical management of UC, the primary approach to achieving remission involves the utilization of 5-ASA and certain small molecular biological agents [54]. Nevertheless, administering these medications frequently results in severe infections or drug resistance among patients, ultimately heightening the likelihood of colectomy [55]. The excessive expression of immune cells, including neutrophils, in the colonic mucosa has been observed to promote the generation of abundant ROS and contribute to oxidative stress. The detrimental feedback loop between ROS and inflammatory responses is a primary driver of the rapid deterioration of UC. Thus, the utilization of antioxidant therapy represents a promising approach to the treatment of UC [56].

Traditional natural enzymes possess a commendable level of safety and exhibit remarkable catalytic activity, rendering them highly suitable for the therapeutic management of inflammation [57]. To illustrate, the extraction of polyphenolic compounds from Nitraria tangutorum Bobr. fruits had been undertaken by researchers to address colitis, and the efficacy of Manganese Superoxide Dismutase (Mn-SOD) treatment of UC had been substantiated via intramuscular administration in a mouse model of colitis [58,59]. However, there are technical limitations concerning the purification and extraction of natural enzymes, resulting in low purity and a complex purification process. Furthermore, since colitis is an inflammation of the gastrointestinal tract, oral administration is the preferred delivery route. Unfortunately, due to the protein nature of enzymes like SOD, their oral administration often leads to rapid degradation.

Consequently, muscle injection is frequently required, resulting in a shorter half-life and reduced efficacy of the therapeutic agents. Artificial nanoenzymes with efficient catalytic activity have been extensively studied as a new therapeutic approach to overcome the limitations and deficiencies of natural enzymes. Therefore, synthesizing a nanoenzyme drug that can efficiently and selectively combat oxidative damage at the inflamed sites in the colon holds significant value for treating UC [60].

In recent years, there has been growing recognition of the significant potential of Pt NPs in biomedical applications [61,62]. Previous research has provided evidence that Pt NPs, particularly those with smaller particle sizes, exhibit diverse antioxidant enzyme activities due to their distinctive electronic structure and size-dependent effects. These inherent properties render Pt NPs a promising and dependable novel material for addressing refractory inflammatory diseases that arise from the accumulation of ROS. Pt NPs are effective in treating neuroinflammatory conditions [63]. Due to their diminutive dimensions and reduced fluidity, nanogels demonstrate a limited capacity for drug encapsulation and inadequate durability as oral drug carriers, and the residual surfactants or unreacted monomers after nanogel preparation can head to toxic effects [64,65].

Nevertheless, many preliminary inquiries have provided evidence for the praiseworthy compatibility with living organisms and the ability to decompose naturally of chitosan/sodium alginate hydrogels. These hydrogels possess a permeable framework and expansive surface area, facilitating efficient adsorption and sustained retention of medicinal compounds. The commendable biocompatibility and biodegradability of chitosan/sodium alginate hydrogels, along with their demonstrated polymerization and disintegration characteristics in varying PH conditions, have been substantiated by numerous preliminary investigations.

Consequently, chitosan/sodium alginate hydrogels emerge as a rational delivery vehicle for nanoenzyme therapy in colitis. Based on the critical role of ROS in the pathological development of UC and the high antioxidant enzyme activity of Pt NPs, colloidal Pt NPs were synthesized using a reduction method for UC prevention in which PVP acted as a stabilizer and dispersant of metal nanomaterials. First, those with a small particle size were synthesized, and the SOD and CAT liked-activities and hydroxyl radical scavenging ability of the Pt NPs (3 ± 1 nm) were confirmed using enzyme activity experiments. These antioxidant enzyme activities are the basis for treating UC with Pt NPs.

ROS are pivotal in regulating essential human growth, adaptation, and various physiological processes [66]. An excessive presence of ROS within specific tissues can disturb the delicate equilibrium of oxidation and result in oxidative harm, thereby intensifying the state of chronic inflammation. Macrophages, being immune cells, generate ROS by releasing proinflammatory factors, thereby initiating inflammatory responses [67,68,69]. Our study assessed Pt NPs’ antioxidant capacity using macrophage RAW 264.7 cells as an inflammatory model. The results showed that Pt NPs effectively prevented oxidative stress and protected cells from oxidative damage. In similar experiments, traditional enzymes exhibited lower efficiency in clearing ROS within inflammatory cells than Pt NPs [59]. Moreover, in preventing a UC mouse model, chitosan/alginate hydrogel-loaded Pt NPs significantly reduced various inflammatory markers, indicating the remarkable ability of Pt NPs to ameliorate UC. Histopathological examination of colon smears from the treated group revealed a notable reduction in neutrophil infiltration in colon tissues.

## 5. Conclusions

The colloidal Pt NPs synthesized in this study possessed strong enzymatic activities similar to SOD and CAT, effectively scavenging •OH radicals. By utilizing chitosan/sodium alginate hydrogels, which possess remarkable biocompatibility and biodegradability, Pt NPs have established a robust basis for effectively eradicating surplus ROS at the inflammatory locus of UC. Pt NPs exhibited remarkable efficacy in mitigating cellular oxidative damage and displaying potent anti-inflammatory properties in models of colitis prevention. Furthermore, Pt NPs did not induce significant systemic toxicity. The findings highlight the considerable potential of Pt NPs as a nanomedicine, providing a novel therapeutic strategy for treating ulcerative colitis.

## Data Availability

All the other data supporting the findings of this study are available within the article and from the corresponding author upon reasonable request.
